# Gender Difference in 2-Year Mortality and Immunological Response to ART in an HIV-Infected Chinese Population, 2006–2008

**DOI:** 10.1371/journal.pone.0022707

**Published:** 2011-08-16

**Authors:** Zhihui Dou, Jiahong Xu, Jin Hua Jiao, Ye Ma, Stephen Durako, Lan Yu, Yan Zhao, Fujie Zhang

**Affiliations:** 1 National Center for AIDS/STD Control and Prevention, Chinese Center for Disease Control and Prevention, Beijing, China; 2 Westat Inc., Rockville, Maryland, United States of America; University of Cape Town, South Africa

## Abstract

**Background:**

Since it was initiated in 2002, the China Free Antiretroviral Treatment (ART) Program has been progressing from an emergency response to a standardized treatment and care system. As of December 31, 2009, a total of 81,880 patients in 31 provinces, autonomous regions, and special municipalities received free ART. Gender differences, however, in mortality and immunological response to ART in this cohort have never been described.

**Objective:**

To understand whether women and men who enrolled in the China National Free ART Program responded equally well to the treatment.

**Methods:**

A retrospective analysis of the national free ART databases from June 2006–December 2008 was performed. HIV-infected subjects who were 18 years or older, ART naïve at baseline, and on a 3TC regimen enrolled in the program from June 1 to December 31, 2006, were included in this study, then followed up to 2 years.

**Results:**

Among 3457 enrolled subjects who met the inclusion criteria, 59.2% were male and 40.8% female. The majority of the subjects were 19–44 years old (77%) and married (72%). Over the full 24 months of follow-up, the mortality rate was 19.0% in males and 11.4% in females (p = 0.0014). Males on therapy for 3–24 months were more likely to die than females (HR = 1.46, 95% CI: 1.04–2.06, p = 0.0307) after adjusting for baseline characteristics. Compared to men, women had higher CD4+ counts over time after initiating ART (p<0.0001).

**Conclusions:**

Our study showed that women had an overall lower mortality and higher CD4+ counts than men in response to ART treatment, which may be attributed to adherence, biological factors, social, cultural and economic reasons. Further study is needed to explore these factors that might contribute to the gender differences in mortality and immunological response to ART.

## Introduction

In the past two decades, advances in antiretroviral treatment (ART) have resulted in dramatic declines in death rates in countries where treatment is available, transforming a once-fatal disease into a manageable chronic illness [1]–[2]. Despite this remarkable achievement, there remain major questions about whether treatment outcomes differ for women and men and what factors may drive such variation. Although a number of studies have examined gender differences in HIV disease progression and in the response to ART, using survival, HIV-1 RNA levels, and lymphocyte subset levels to assess response to treatment, the findings have differed with regard to the association of gender with these measures. Early studies showed a more rapid clinical progression in women, which was attributed to the delay in starting ART and to other gender-related conditions such as discrimination, violence, and stigma [3]. More recently, natural history cohorts observed that early in infection, women have significantly lower amounts of the virus in their blood than do men but suffer the loss of immune cells and develop AIDS just as swiftly as men [4]–[6]. A cohort study of 2196 HIV infected treatment-naïve adults conducted in South Africa reported that gender was not significantly associated with survival after adjusting for baseline clinical and immunovirological status [7]. Conversely, several studies have found evidence that gender was associated with response to ART [8]–[11]. Given that HIV/AIDS has affected more women worldwide than any other life threatening infectious disease [12] and that half of the estimated 30.8 million HIV-infected adults worldwide are women [13], it is critical to have a better understanding of the gender influence in survival and immunological responses to ART. As more and more women are impacted by the HIV epidemic in China [14], it has also become of utmost importance to understand whether women and men respond differently to ART treatment.

In response to the growing HIV epidemic in China, the Chinese government responded in 2002 with a national ART program called the National Free Antiretroviral Therapy Program (NFATP), which provides antiretroviral (ART) drugs free to those most in need [15]. To monitor and evaluate the success of the NFATP, China also established a Free ART Database in 2004 to collect demographic, treatment, and clinical care information on all patients participating in the NFATP [16]. As of December 2009, the Free ART Database had data from 81,880 patients in 31 provinces and autonomous regions who had received ART through the NFATP [17]. A recent analysis of factors associated with treatment outcome in patients registered in this database suggested a positive association of female gender with good treatment outcome [18], but factors that might explain this association were not explored further. In this study, we extend the previous analysis to examine other factors that might explain the gender-related difference in treatment outcome, using a subset of the NFATP cohort that were treatment naïve when entering the program, received initial treatment with a HAART regimen containing 3TC (Lamivudine), and had at least two-year potential follow-up time since initiating therapy.

## Methods

This analysis was approved by the institutional review board (IRB) of the National Center for AIDS/STD Control and Prevention (NCAIDS), China CDC. Per the IRB review, individual informed consent was waived because this analysis used currently existing data collected during the course of routine treatment and care and data were reported in aggregate without use of individual identifying information.

### Study Design and Population

As described previously [17], [18], the NFATP enrolled HIV-infected subjects who met the national ART treatment guidelines, which includes CD4+ T-cell count <200 cells/uL (<350 cells/uL in the revised edition in 2008) or World Health Organization (WHO) stage III or IV disease. After enrollment, subjects were followed at week 2, months 1, 2, 3, and then every 3 months thereafter. Clinical information - including clinical signs and symptoms, self-reported adherence, and laboratory test results – were collected at the time of treatment initiation and at each subsequent follow-up visit. WHO staging and TB data were not collected. The local health providers collected these data on site, using standard case report forms and then transferred these data to the national database.

A retrospective analysis was performed on subjects enrolled in the NFATP who had data in the Free ART Database from June 2006–December 2008. Eligible subjects had to be enrolled in the NFATP between June 1–December 31, 2006 to allow for two years of follow-up, at least 18 years of age, ART-naïve at time of enrollment, and received a HAART regimen that included 3TC (Lamivudine) as their initial therapy including D4T +3TC+NVP; D4T +3TC +EFV; AZT+3TC +NVP; or AZT+3TC +EFV. A 3TC regimen was required because it became readily available in China in 2005, has lower toxicity than other regimens, and has become the norm for initial therapy in China.

After enrollment, subjects were followed up to 2 years to observe ART response including immunological function (CD4+ counts), side effects to the ART, adherence and mortality. Data from enrollment to visits through December 31, 2008 were included in the analysis to allow the last-enrolled subjects to have up to 2 years of follow-up after treatment initiation. For those who were enrolled earlier, follow-up visits after 24 months were eliminated from this analysis. Subjects with missing gender, missing treatment initiation date, first-line regimen with less than three single drugs or including a combination of AZT+D4T (not allowed due to drug interaction) were excluded from this study. A total of 4791 subjects enrolled into the NFATP from June 1 to December 31, 2006. Of those, 3457 subjects were eligible for this study. When subjects were lost to follow-up or terminated treatment during the two-year follow-up, they were censored from the study, and the length on study was defined as the date of enrollment to the date of the last available visit. Subjects who transferred from one site to another within the program were considered as adherent to study visits.

### Study Measures

Demographic and baseline characteristics were obtained, including gender, age, marital status, mode of HIV transmission, signs and symptoms at enrollment, and ART regimens. In this population, the HIV transmissions were mainly through commercial blood donations, noted as the former plasma donor (FPD); intravenous drug use (IDU); and sexually transmitted infection (STI). Laboratory test data included absolute CD4+ cell counts, hemoglobin and liver function, which were collected at baseline and follow-up visits. CD4+ T-cell counts were collected during at least one of the follow-up visits (months 3, 6, 12, 18 and 24). When the CD4+ count was not available for a scheduled follow-up visit, an alternative CD4+ count within ±1.5 months of the scheduled visit time window was used for the analysis. HIV viral load was not available for analysis as a treatment outcome, since the lack of local resources during this time period prevented testing for viral load in most subjects. Based on Division of AIDS Table for Grading the Severity of Adult and Pediatric Adverse Events (GSAPAE) for HIV positive subjects only, hemoglobin was categorized as three groups (normal or grade 0: >100 g/dL; grade 1 or 2: 75–100 g/dL; and grade 3+: <75 g/dL), and liver function tests including AST and ALT were categorized as ≥100 U/L vs. <100 U/L (2.5×ULN). Self-reported current signs and symptoms of HIV infection such as fever, diarrhea, rash, fatigue, weight loss and lymphadenopathy, were collected at baseline. Self-reported medication adherence and fourteen self-reported adverse events (including appetite change, vomiting, sleeping difficulties, abdominal pain, dry skin, skin rush, numb limbs, pain limbs, fatigue, body shape change, hair loss, vision change, headache, and vivid dreams) were also collected at follow-up visits. The self-reported medication adherence was collected as an ordinal variable with 0, 1–5, 6–10, 11–15, 16–20 and >20 times missing doses a month, but was redefined as a binary measure (100% vs. <100%) for this study. When a subject did not report having any missing dose, this subject was considered as perfectly adherent to ART medication (100%); otherwise, the subject was considered as less than 100% adherent (a subject reported missing at least one dose during follow-up).

### Statistical Analyses

Chi-square was used to examine the differences in proportion of deaths between males and females. Kaplan-Meier survival curves and Log-Rank test were used to display and test gender differences in mortality over 2 years after initiating ART treatment. Univariate and multivariable Cox proportional hazard models were constructed to examine the gender effect on mortality. The demographic and baseline characteristics as well as medication adherence were included as the potential risk factors. The proportional hazard (PH) assumption over time was tested using a graphic approach and time-dependent variables where appropriate. When the PH assumption was not met because of a time/gender interaction, PH models were constructed separately for the subset of subjects who were in the program for up to three months (0–3 months) and for the larger subset who were in the program for up to 24 months (3–24 months). Another subset analysis was conducted among those who had CD4+ counts at baseline and at least three test results available after enrollment. The mixed linear model (MIXED) with repeated measurements was used to examine the effect of gender and other potential covariates on CD4+ count change over time. The unstructured covariance was assumed for the covariance structure on CD4+ counts. Model fit statistics (e.g. AIC, BIC, etc) were used to choose the best covariance structure.

## Results

### Demographic and Baseline Characteristics

**Figure 1:**
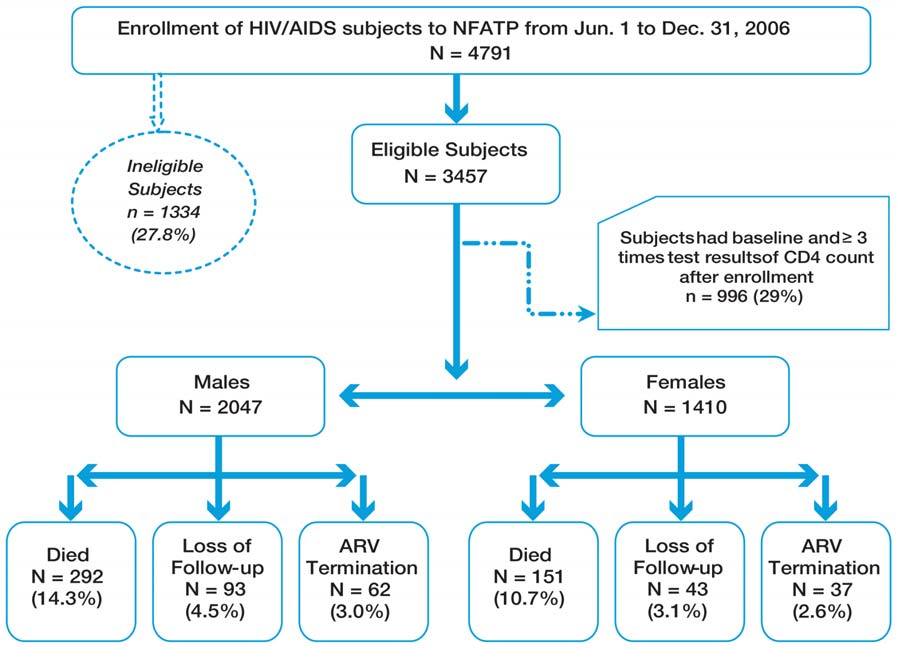
Subjects Attrition over Time. Ineligible subjects: 1. ART initiation regimens were not D4T+3TC+NVP, D4T+3TC+EFV, AZT+3TC+NVP, AZT+3TC+EFV: n = 806; 2. ART termination or death date were prior to ART initiation date (data entry error): n = 10; 3. Not ART naïve at enrollment: n = 423; 4. Age <18 years old or missing at ART initiation: n = 82; 5. Gender missing: n = 13.

Among 3457 enrolled subjects who met the inclusion criteria, 59.2% were male and 40.8% females (Table 1). Majority of the subjects were 19–44 years old (77% for both genders), married (68% for males and 77% for females). Among all transmission routes, sexual transmission was the most frequent for both males and females (33% for males and 44% for females), followed by IDU for males (30%) and FPD for females (41%). Over two-thirds of all subjects had experienced signs/symptoms of HIV infection, but men had significantly more occurrences (89% vs. 83%, p<0.001). The median baseline CD4+ counts were lower for men compared with women (96 vs. 131 cells/uL, p<.0001) and more men than women had CD4+ counts <50 cells/uL at enrollment (33.5% vs. 25.5%, p<.0001). More females had a grade 1 or higher abnormal hemoglobin (26%) but more males had abnormal liver function (≥100 U/L) at baseline (8%). Among 3457 patients, 136 (3.9%) were lost to follow-up and 99 (2.9%) terminated ART (Figure 1). No subjects transferred out of the program. The median follow-up time for both male and female subjects was 21.3 months.

**Table 1:** Demographic and HIV/AIDS Related Characteristics at Baseline by Gender.

	Total	Male	Female	
	(N = 3457)n (%)^1^	(N = 2047)n (%)^1^	(N = 1410)n (%)^1^	p value^2^
Gender				
Male	2047 (59.20)			
Female	1410 (40.80)			
Age (years)				
Mean (std. dev.)	38.42 (10.17)	38.75 (10.15)	37.94 (10.17)	
Median (IQR)	37 (31–44)	37 (31–44)	37 (31–43)	0.0544
19–44	2671 (77.35)	1574 (77.01)	1097 (77.86)	0.1007
45–59	642 (18.59)	375 (18.35)	267 (18.95)	
≥60	140 (4.05)	95 (4.65)	45 (3.19)	
Marital Status				
Single	486 (14.06)	397 (19.39)	89 (6.31)	<.0001
Married	2475 (71.59)	1385 (67.66)	1090 (77.30)	
Divorced	214 (6.19)	146 (7.13)	68 (4.82)	
Widowed	279 (8.07)	116 (5.67)	163 (11.56)	
Unknown	3 (0.09)	3 (0.15)	0 (0.00)	
Transmission Routes				
FPD	1167 (33.76)	586 (28.63)	581 (41.21)	<.0001
IDU	720 (20.83)	620 (30.29)	100 (7.09)	
STI	1299 (37.58)	676 (33.02)	623 (44.18)	
Other/Unknown	271 (7.84)	165 (8.06)	106 (7.52)	
Any Past and/or Current Signs/Symptoms at Enrollment				
Yes	2993 (86.58)	1820 (88.91)	1173 (83.19)	<.0001
No	464 (13.42)	227 (11.09)	237 (16.81)	
Baseline AZT Regimen				
Yes	734 (21.23)	434 (21.20)	300 (21.28)	0.9578
No	2723 (78.77)	1613 (78.80)	1110 (78.72)	
CD4+ Counts (cells/uL)				
Mean (std. dev.)	125.00 (106.36)	117.08 (104.84)	136.73 (107.54)	
Median (IQR)	109 (36–190)	96 (31–180)	131 (47–200)	<.0001
≥200	674 (21.66)	359 (19.32)	315 (25.12)	<.0001
50–199	1496 (48.07)	877 (47.20)	619 (49.36)	
<50	942 (30.27)	622 (33.48)	320 (25.52)	
Hemoglobin				
Normal or Grade 0	2580 (80.65)	1607 (84.94)	973 (74.45)	<.0001
Grade 1 or 2	504 (15.75)	235 (12.42)	269 (20.58)	
≥Grade 3	115 (3.59)	50 (2.64)	65 (4.97)	
Liver Function (AST/ALT)				
≥100 U/L	179 (5.74)	140 (7.52)	39 (3.10)	<.0001
<100 U/L	2942 (94.26)	1721 (92.48)	1221 (96.90)	

1 The group frequencies may not sum to total due to missing values.

2 *P*-values are from Chi-square test for categorical variables and non-parametric test for continuous variables.

### Two-Year Mortality

**Figure 2:**
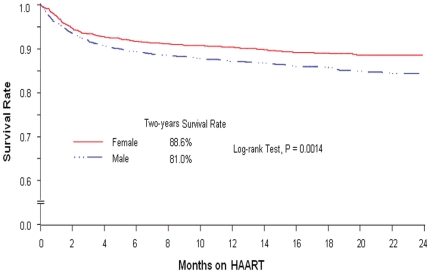
Two-Years Survival on ART Treatment by Gender among 3457 Subjects.

Overall, 443 (12.8%) of 3457 subjects were died in the first two years after initiating ART treatment. Among them, 151 (10.7%) of 1410 females and 292 (14.3%) of 2047 males were died. A significantly greater death incidence from Kaplan-Meier (KM) estimates in males 24 months after enrollment was detected when compared with female subjects (19.0% vs. 11.4%, p = 0.0014). The estimated two-year overall survival rate, unadjusted for covariates, was 84.3%; two-year survival rate for females was significantly higher than for males (Figure 2, 88.6% vs. 81.0%, p = 0.0014).

**Figure 3:**
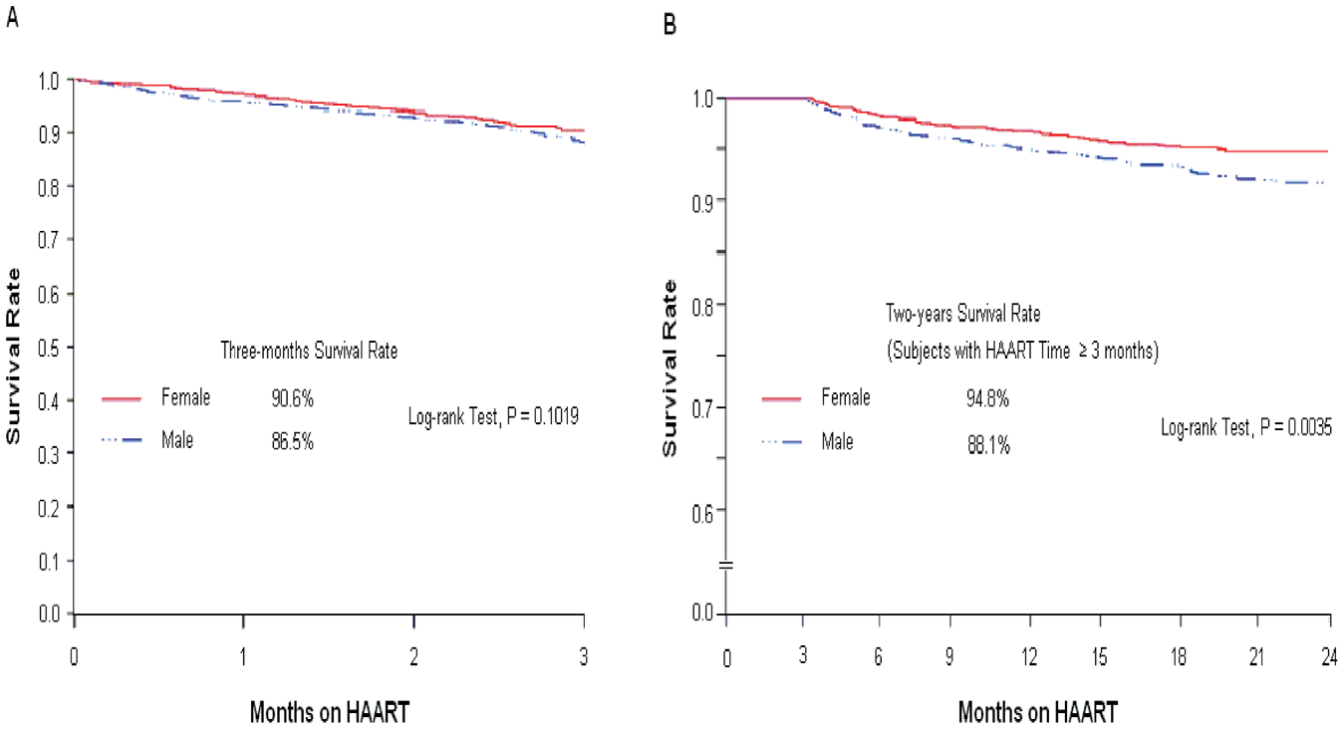
Early and After 3-Months Survival after on ART Treatment by Gender.

We observed that mortality was higher during the early phase of the treatment (0–3 months). Of the 443 deaths observed in the first two years, about 248 (56%) occurred in the first three months after ART initiation. In addition, for multivariate analysis adjusting for covariates, the PH assumption of constant hazard was not satisfied over the entire 24-month period. There was a time/gender interaction (p = 0.0411), with the hazard rate for females changing over time. Therefore, the separated analyses were further performed: those in the program for 0–3 months and those in the program for greater than 3 months. The PH assumption was satisfied for these two separate groups. There was no difference in survival between males and females among those early deaths (Figure 3, Panel A). In contrast, for the other 195 (44%) subjects who died after three months, the survival rate for females was significantly higher than for males (Figure 3, Panel B). The results from the multivariable Cox models showed that there was no evidence of gender effect on early mortality (p = 0.0940) but males were at greater risk of dying after being on treatment for at least three months (HR = 1.46, 95% CI: 1.04–2.06, p = 0.0307) after adjusting for HIV transmission routes, baseline and current signs and symptoms, and baseline CD4+ counts (Table 2). Although gender was not associated with the mortality during the early stage of treatment (0–3 months), there was a strong association with mortality for two other covariates (having baseline signs/symptoms and lower CD4+ counts) in this early period even stronger than later in treatment (3–24 months).

**Table 2:** Relationship of HIV/AIDS Mortality with Demographic and HIV/AIDS Related Characteristics.

	Overall (N = 3457)		On Treatment for <3 Months (N = 456)		On Treatment for 3–24 Months (N = 3001)	
		Unadjusted	Unadjusted	Adjusted	Unadjusted	Adjusted
	Died/n (%)	HR (95% CI)^1^^,^ 2	HR (95% CI)^1^	HR (95% CI)^1^^,^ 3	HR (95% CI)^1^	HR (95% CI)^1^^,^ 3
Gender						
Male	292/2047 (14.26)	1.38 (1.13–1.68)	1.12 (0.86–1.45)	1.31 (0.95–1.81)	1.56 (1.15–2.11)	1.46 (1.04–2.06)
Female	151/1410 (10.71)	1.00	1.00	1.00	1.00	1.00
Age (years)						
19–44	330/2671 (12.35)	1.00	1.00		1.00	
45–59	86/642 (13.40)	1.05 (0.83–1.33)	1.34 (0.95–1.88)	NS	1.26 (0.90–1.77)	NS
≥60	27/140 (19.29)	1.64 (1.11–2.43)	1.22 (0.74–2.01)		1.47 (0.77–2.79)	NS
Marital Status						
Single	55/486 (11.32)	1.00	1.00		1.00	
Married	309/2475 (12.48)	1.03 (0.78–1.38)	1.16 (0.79–1.71)	NS	0.97 (0.64–1.49)	NS
Divorced	44/214 (20.56)	1.87 (1.26–2.78)	1.02 (0.61–1.71)	NS	1.54 (0.82–2.88)	NS
Widowed	35/279 (12.54)	1.05 (0.69–1.61)	1.44 (0.80–2.60)	NS	1.08 (0.58–2.00)	NS
Transmission Routes						
FPD	169/1167 (14.48)	1.00	1.00	1.00	1.00	1.00
IDU	96/720 (13.33)	1.00 (0.78–1.28)	0.40 (0.27–0.58)	0.54 (0.35–0.84)	1.28 (0.90–1.80)	1.27 (0.87–1.85)
STI	144/1299 (11.09)	0.78 (0.62–0.97)	0.76 (0.57–1.02)	0.78 (0.54–1.12)	0.57 (0.40–0.82)	0.57 (0.39–0.84)
Other/Unknown	34/271 (12.55)	0.91 (0.63–1.31)	0.93 (0.58–1.46)	0.91 (0.54–1.54)	0.63 (0.33–1.18)	0.55 (0.27–1.12)
Any Past and/or Current Signs/Symptoms at Enrollment						
Yes	428/2993 (14.30)	4.79 (2.86–8.01)	5.66 (1.81–17.66)	5.71 (1.39–23.39)	2.66 (1.49–4.78)	1.91 (1.02–3.57)
No	15/464 (3.23)	1.00	1.00	1.00	1.00	1.00
CD4+ Counts						
≥200	26/674 (3.86)	1.00	1.00	1.00	1.00	1.00
50–199	145/1496 (9.69)	2.51 (1.66–3.82)	3.50 (1.60–7.66)	3.48 (1.58–7.67)	1.98 (1.20–3.25)	1.95 (1.18–3.21)
<50	188/942 (19.96)	5.70 (3.78–8.59)	5.66 (2.63–12.18)	4.60 (2.11–10.06)	3.14 (1.90–5.21)	3.04 (1.82–5.07)
Hemoglobin						
Normal or Grade 0	263/2580 (10.19)	1.00	1.00		1.00	
Grade 1 or 2	100/504 (19.84)	2.13 (1.69–2.69)	1.35 (1.00–1.82)	NS	1.59 (1.10–2.29)	NS
≥Grade 3	32/115 (27.83)	3.25 (2.25–4.69)	2.14 (1.37–3.35)	NS	1.91 (0.97–3.76)	NS
Liver Function: AST/ALT						
≥100 U/L	37/179 (20.67)	1.95 (1.39–2.74)	1.35 (0.86–2.10)	NS	1.83 (1.08–3.11)	NS
<100 U/L	341/2942 (11.59)	1.00	1.00		1.00	
Self-reported ART Adherence						
100%	267/2444 (10.92)	1.18 (0.92–1.52)	1.14 (0.75–1.72)	NS	0.91 (0.66–1.25)	NS
<100%	78/819 (9.52)	1.00	1.00		1.00	

1 HR: hazard ratio; NS: the covariate was either statistically insignificant (not entered into the initial full multivariable Cox model) or deleted during the model selection procedure; the proportional hazard assumption was tested using graphic approach (univariate model only) and time-dependent variable (both univariate and multivariable models).

2 The PH-assumption for the univariate model (gender) among all subjects was satisfied (p = 0.3000) but was violated for the overall multivariate final model (p = 0.0411).

3 The PH-assumption was satisfied for 0–3 months (p = 0.6228) and 3–24 months (p = 0.4097) final multivariate models. The R-square for the final multivariable models are 86% for 0–3 months and 12% for 3–24 months.

### CD4+ T-Cell Count Response to ART Treatment

CD4+ T-cell count was examined to assess gender differences in immunological response to ART. Due to limited resources, CD4+ counts were only done at a few visits for some subjects. Thus, we were unable to adjust for CD4+ cell count change over time in the multivariate proportional hazard models described earlier. We were, however, able to make inferences on CD4+ change over time for subjects who had baseline and at least 3 follow-up CD4+ count test results. The results from the multivariable mixed model with repeated measurements indicated that the average CD4+ cell count change over time in males was significantly lower than in females (p<0.0001) after adjusting for other factors including being older, infected through FPD, having past and current signs and symptoms, lower baseline CD4+ counts, without abnormal liver function, and less than 100% ART adherence (Table 3).

**Table 3:** Impact of Gender and Other Factors on Immunology Responses to ART Treatment Over Time among 996 Subjects.

	Unadjusted		Adjusted	
	Est. Coef. (Std. Err.)†	p-value	Est. Coef. (Std. Err.)†	p-value
Gender				
Male	−33.34 ( 4.79)	<.0001	−22.11 ( 4.79)	<.0001
Female	0.00		0.00	
Age (years)				
≥60	−42.72 (12.53)	0.0007	−30.70 (12.81)	0.0166
45–59	−25.22 ( 5.93)	<.0001	−15.07 ( 5.77)	0.0091
19–44	0.00		0.00	
Marital Status				
Widowed	−0.81 (10.75)	0.9397	NS	
Divorced/Separated	7.46 (11.68)	0.5230	NS	
Married/Live Together	−13.75 ( 6.90)	0.0465	NS	
Single	0.00			
Transmission route				
Other	15.83 ( 8.75)	0.0704	33.43 ( 8.50)	<.0001
STI	20.71 ( 5.59)	0.0002	30.26 ( 5.63)	<.0001
IDU	46.35 ( 7.51)	<.0001	42.40 ( 7.74)	<.0001
FPD	0.00		0.00	
Any Past and/or Current Signs/Symptoms				
Yes	−59.89 ( 6.19)	<.0001	−14.36 ( 6.06)	0.0179
No	0.00		0.00	
Baseline CD4+ Counts (cell/uL)				
<50	−212.6 ( 6.44)	<.0001	−205.1 ( 7.04)	<.0001
50–199	−111.8 ( 5.81)	<.0001	−104.4 ( 6.14)	<.0001
≥200	0.00		0.00	
Hemoglobin				
Grade 3 & Grade 4	−61.59 (14.63)	<.0001	NS	
Grade 1 & Grade 2	−61.44 ( 6.67)	<.0001	NS	
Normal & Grade 0	0.00			
Liver Function (AST/ALT)				
Yes	27.74 (12.24)	0.0235	24.95 (11.08)	0.0244
No	0.00		0.00	
Self-Reported ART Adherence				
100%	11.87 ( 5.51)	0.0313	11.46 ( 5.20)	0.0277
<100%	0.00		0.00	

† Est. Coef.: estimated coefficient; Std. Err.: standard error.

### Self-Reported Adverse Events

We attempted to examine the self-reported adverse events at the selected follow-up visits (months 3, 6, 12, 18, and 24). When the events were missing at a specific month, the imputation was made by using an available data point within ±1.5 months around that specific follow-up visit. The results indicated that females tended to report more adverse events at most follow-up visits. For example, the overall proportion of self-reporting at least one adverse event among females was significantly higher than males at 3 months (28.7% vs. 23.5%, p = 0.0019), 6 months (23.5% vs. 19.0%, p = 0.0053), and 12 months (18.6% vs. 14.7%, p = 0.0095).

## Discussion

Our current study on this homogenously-treated population confirmed that females have better survival at 2 years on ART than men do. Compared to men, females had overall better immunological response overtime while on ART. Our findings are consistent with others. Hall et al [8], using data from the CDC national HIV/AIDS reporting system from 1996 to 2001, observed that the risk for progression from HIV to AIDS or death was greater for nonwhites, men, and older persons compared with whites, women, and younger persons, respectively. Collazos et al [9] found that women had better clinical and viro-immunological responses than men and concluded that gender played a small but significant role in outcome. An observational study conducted in the US reported that lower pre-therapy CD4+ cell count, younger age, and female sex were all factors associated with increased CD4+ cell count gains from month 3 to year 4 of ART [3]. A recent study performed in Uganda and Zimbabwe on the virologic and immunological outcomes of 300 HIV-infected adults treated with nucleoside reverse transcriptase inhibitors (NRTIs), women showed a 2.3 fold higher rate of virologic suppression compared with men after 48 weeks on ART [11].

Our finding that mortality did not differ by gender in the first 3 months on treatment may suggest that a certain proportion of patients entered therapy at a late enough stage of disease progression that there is little opportunity for therapy to work. This hypothesis is supported by our findings that mortality was much higher in the first 3 months and that it was strongly associated with greater baseline signs/symptoms and lower baseline CD4+ counts. After 3 months the hazard rate of females diverged from that of males.

There are a number of possible explanations for our findings: (1) biologically better response to the drugs; (2) better adherence to treatment; and (3) other factors such as co-morbid conditions, general health status, and psychosocial differences. Our analysis, together with the recent findings of Rotger, et al. [19], Heath, et al. [20], and Van, et al. [21], suggest that there may be a real difference between females and males in biological response to the ARTs. Because the NFATP data are from a registry, we had limited items that measured co-morbidity, general health status, and psychosocial factors. While a higher proportion of males had abnormal liver function, suggesting possible co-infection with hepatitis B or C, this did not explain the treatment response difference. Males were also much more likely to have been infected through injection drug use, suggesting both poorer overall health status and poorer psychosocial status. Our results suggest that there may be very different factors influencing early and later mortality, which merits further detailed laboratory investigation, since it might reveal genetic or other biological markers that would improve the personalization of treatment regimens for both women and men.

Our study has limitations because of the nature of the information that was retrospectively obtained from the Free ART database from 31 provinces of China, certain levels of recall bias and measurement variations may be introduced in data collection. Second, HAART initiation relies on both WHO staging and CD4+ count; however, WHO stage data was not collected until 2010. Although signs/symptom data were analyzed, this may not truly reflect WHO stage. Third, we were not able to analyze viral load response because it was infrequently collected in the field. Fourth, the CD4+ analysis was restricted to the subjects who had CD4 tests 3 times or more after the treatment initiation. Although the demographic characteristics (e.g. gender, age, and marital status) from this subset were comparable to the rest of the population, this subset may not truly represent the entire population of subjects who were included in the analysis. Fifth, self-reported treatment adherence data may not be completely accurate, and it is possible that some treatment response differences might still be attributable to difference in adherence. Finally, we had limited information collected on other possible explanatory factors, such as co-morbidities, general health, and psychosocial status.

Our findings of lower mortality rates among females after 3 months on ART and better immunological response among females in a subset of our cohort demonstrate a difference in outcomes between men and women in the first 2 years of treatment with ART. Given the limitation of data it is unclear whether this is due to environmental, psychological, or biological factors. Further controlled prospective data collection should be undertaken to determine whether biological factors related to gender play an important role in determining outcomes to treatment. Further prospective studies within our own treatment cohort are necessary to explore other possible explanatory factors such as adherence, co-morbidities, general health status and psychosocial factors.

Recommendations: Viral load is a gold standard to evaluate whether patients are virologically suppressed after ART. It is important to collect viral load data at certain time points after ART. Intensive adherence counseling for patients on failing regimens would increase the optimization of first line regimens and save money in the long term.
